# Assessing Public Interest in Mammography, Computed Tomography Lung Cancer Screening, and Computed Tomography Colonography Screening Examinations Using Internet Search Data: Cross-Sectional Study

**DOI:** 10.2196/53328

**Published:** 2025-03-11

**Authors:** Zachary D Zippi, Isabel O Cortopassi, Rolf A Grage, Elizabeth M Johnson, Matthew R McCann, Patricia J Mergo, Sushil K Sonavane, Justin T Stowell, Brent P Little

**Affiliations:** 1 Florida International University College of Medicine, Miami, FL, United States; 2 Mayo Clinic College of Medicine and Science, Mayo Clinic Florida, 4500 San Pablo Road, Jacksonville, FL, 32224, United States, 1 904-953-0853

**Keywords:** lung cancer, lung cancer screening, breast cancer, mammography, colon cancer, CT colonography, Google search, internet, Google Trends, imaging-based, cancer screening, search data, noninvasive, cancer, CT, online, public awareness, big data, analytics, patient education, screening uptake

## Abstract

**Background:**

The noninvasive imaging examinations of mammography (MG), low-dose computed tomography (CT) for lung cancer screening (LCS), and CT colonography (CTC) play important roles in screening for the most common cancer types. Internet search data can be used to gauge public interest in screening techniques, assess common screening-related questions and concerns, and formulate public awareness strategies.

**Objective:**

This study aims to compare historical Google search volumes for MG, LCS, and CTC and to determine the most common search topics.

**Methods:**

Google Trends data were used to quantify relative Google search frequencies for these imaging screening modalities over the last 2 decades. A commercial search engine tracking product (keywordtool.io) was used to assess the content of related Google queries over the year from May 1, 2022, to April 30, 2023, and 2 authors used an iterative process to agree upon a list of thematic categories for these queries. Queries with at least 10 monthly instances were independently assigned to the most appropriate category by the 2 authors, with disagreements resolved by consensus.

**Results:**

The mean 20-year relative search volume for MG was approximately 10-fold higher than for LCS and 25-fold higher than for CTC. Search volumes for LCS have trended upward since 2011. The most common topics of MG-related searches included nearby screening locations (60,850/253,810, 24%) and inquiries about procedural discomfort (28,970/253,810, 11%). Most common LCS-related searches included CT-specific inquiries (5380/11,150, 48%) or general inquiries (1790/11,150, 16%), use of artificial intelligence or deep learning (1210/11,150, 11%), and eligibility criteria (1020/11,150, 9%). For CTC, the most common searches were CT-specific inquiries (1800/5590, 32%) or procedural details (1380/5590, 25%).

**Conclusions:**

Over the past 2 decades, Google search volumes have been significantly higher for MG than for either LCS or CTC, although search volumes for LCS have trended upward since 2011. Knowledge of public interest and queries related to imaging-based screening techniques may help guide public awareness efforts.

## Introduction

Worldwide, an estimated 20 million new cancer diagnoses and 9.7 million deaths occurred in 2022. The 3 most common types of newly diagnosed malignancies were lung cancer (12.4%), female breast cancer (11.6%), and colorectal cancer (9.6%). Lung cancer and colorectal cancer were the most common causes of cancer-related mortality, with breast cancer in fourth place after liver cancer [1]. Noninvasive imaging, such as mammography (MG), low-dose computed tomography (CT) for lung cancer screening (LCS), and CT colonography (CTC), plays important roles in the early detection of the most common cancer types and has demonstrated efficacy in reducing cancer-related and all-cause mortality rates [2,3]. Encouraging results from large screening trials in several countries [4-6] have driven global interest in imaging-based lung, breast, and colorectal screening [7-12] and have prompted efforts to initiate and optimize screening worldwide [13-15].

A 2013 Pew Research Center survey found that 72% of adults had pursued web-based health information over the past year, with 77% performing an initial search using an internet search engine [16]. Analysis of internet search volumes for topics related to these imaging examinations may identify opportunities for improved patient outreach and education. Google search trends have been shown to correlate with both epidemiological data and public interest [17-19]. However, few studies have looked at general search volumes for each screening method [20-22], and to our knowledge no publication has documented a detailed topic-level analysis comparing all 3 types of image-based screening.

## Methods

### Overview

Google Trends was used to assess long-term variation in worldwide relative search volumes for the terms “mammography,” “lung cancer screening,” and “CT colonography” for the period January 1, 2004 (the earliest date for which Google Trends data were retrievable) to April 1, 2023. Google was chosen, as it is the most frequently used internet search engine, consistently capturing more than 80% of the worldwide internet search market [23].

Keyword tool (keywordtool.io), a commercial search engine tracking product, was used to query average volumes of monthly Google searches in English worldwide for the period May 1, 2022, to April 30, 2023 [24]. Keyword tool uses the output of the Google autocomplete tool to extract the most common entries at the search prompt, which made it optimal to use for this study design [24]. All questions and question-like queries entered at the Google search prompt relating to the terms “mammography,” “lung cancer screening,” and “CT colonography” were extracted. Two of the authors (ZDZ and BPL) used an iterative process to agree upon a list of thematic categories for queries. Search results with at least 10 monthly searches were independently assigned to the most appropriate category by the 2 graders, with disagreements resolved by consensus.

### Data Analysis

Search volumes for each screening type were plotted as normalized values (relative search volumes comprising the format of Google Trends data output), with 100 representing the highest relative search volume and 1 representing the lowest, with 0 representing a search volume of zero or insufficient search volume to calculate. Average monthly search volumes for each category were grouped by screening modality and presented in tabular format as the number of searches and as a percentage of total searches for the corresponding modality.

## Results

**Figure 1.**
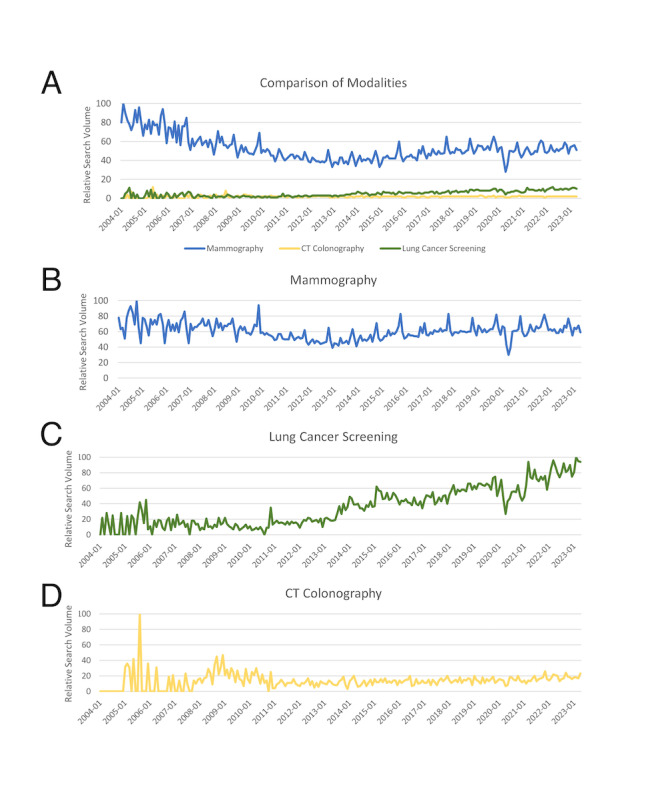
Relative worldwide monthly Google search volumes for imaging-based screening examinations. Graph A compares the relative average worldwide monthly search volumes for mammography, lung cancer screening, and CT colonography from January 1, 2004, to April 1, 2023. Graphs B, C, and D illustrate search volumes for the modalities taken independently. All data were obtained from Google Trends for worldwide searches in English. CT: computed tomography.

A plot of Google Trends data from 2004 to 2023 comparing MG, LCS, and CTC Google searches is shown in Figure 1. The median 20-year relative search volume was 50 (SD 13; IQR 43-57) for MG, 5 (SD 3; IQR 2-7) for LCS, and 2 (SD 1.2; IQR 1-2) for CTC. On average, the search frequency for LCS was only one-tenth of that for MG, and CTC search volume was only one–twenty-fifth of MG search volume. Search volumes for LCS steadily increased from 2011 to 2023, while searches for MG and CTC plateaued.

The relative search volumes for MG alone (Figure 1) across the period ranged from 30 to 100 (median 60; IQR 54-67); there was an overall decrease in relative search volume from the high in 2004 (100) to 2013 (39), followed by an overall increase from 2013 to 2019 (82), with the nadir in 2020 (30) corresponding to the onset of the COVID-19 pandemic. Relative search volumes for LCS alone ranged from 0 to 100; there was an overall decrease in relative search volumes from 2005 (90) to 2010 (0), and a subsequent uptrend after 2011, culminating in a high of 100 in 2019. Relative search volumes for CTC alone ranged from 0 to 100 with the highest volume in 2005, with a secondary peak in 2008 (47), and a plateau from 2010 to 2023 with an average of 14.

Topic analysis using Keyword tool data for the period from May 1, 2022, to April 30, 2023, showed that MG-related topics had the highest total monthly search volume (621,810 searches/month), followed by those related to LCS (23,250 searches/month), and CTC (17,690 searches/month). From these searches, a total of 751 queries classified as questions or question-like queries by the Keyword tool interface were extracted as follows: 442 for MG, 178 for LCS, and 131 for CTC. Of these, 332 had a search volume of >10 per month. The reviewers identified 39, 14, and 11 thematic categories for MG, LCS, and CTC, respectively. The most common categories of queries for MG were nearby screening locations (60,850/253,810, 24%), general inquiries (52,460/253,810, 21%), pain associated with screening (28,970/253,810, 11%), and eligibility criteria (ages) (16,160/253,810, 6%) (Table 1). For LCS, the most frequent categories of queries were CT-specific inquiries (5380/11,150, 48%), general inquiries (1790/11,150, 16%), artificial intelligence (AI) or deep learning use in lung screening (1210/11,150, 11%), screening eligibility criteria (ages or pack-years) (1020/11,150, 9%), and nearby screening locations (750/11,150, 7%) (Table 1). The most common categories of queries related to CTC were CT-specific inquiries (1800/5590, 32%), screening procedural details (1380/5590, 25%), performance compared with colonoscopy (870/5590, 16%), and screening preparation (such as colon preparation and sedation) (720/5590, 13%) (Table 1). Examples of the most common queries for each image-based screening are presented in Table 2.

**Table 1.** Average monthly search volumes for imaging-based screening examinations by search topic category. The table displays the average monthly Google search volumes for imaging-based screening examinations from May 1, 2022, to April 30, 2023. All data were obtained from Keyword tool (keywordtool.io) for worldwide searches performed in English. The top 10 thematic categories of search topics are listed for each modality.

Imaging-based cancer screening search query categories	Average monthly search volume, n (%)
MG^a^ (n=253,810)	
Nearest screening locations	60,850 (24)
General inquiries	52,460 (21)
Pain associated with screening	28,970 (11)
Cancer imaging characteristics	28,890 (11)
Screening eligibility criteria	16,160 (6)
Screening procedural details	11,810 (5)
Comparison of screening modalities (MG vs MRI^b^)	9120 (4)
General screening inquiries	8180 (3)
Opportunities for no-cost screening	6810 (3)
Breast tomosynthesis	6770 (3)
Other categories (combined)	23,790 (9)
LCS^c^ (n=11,150)	
CT^d^-specific inquiries	5380 (48)
General inquiries	1790 (16)
Artificial intelligence and deep learning in LCS	1210 (11)
Screening eligibility criteria	1020 (9)
Nearest screening locations	750 (7)
Opportunities for no-cost screening	420 (4)
Screening procedural details	230 (2)
Insurance coverage	150 (1)
LCS trials	90 (0.8)
Imaging accuracy	40 (0.4)
Other categories (combined)	70 (0.6)
CTC^e^ (n=5590)	
CT-specific inquiries	1800 (32)
Screening procedural details	1380 (25)
Imaging modality comparison (colonoscopy vs CTC)	870 (16)
Prescreening procedural preparation	720 (13)
Coding (*ICD*^f^/CPT^g^)	230 (4)
Nearest screening locations	230 (4)
Pain associated with screening	220 (4)
Opportunities for no-cost screening	60 (1)
Diagnostic capabilities	60 (1)
Insurance coverage	10 (0.2)
Other categories (combined)	10 (0.2)

a MG: mammography.

b MRI: magnetic resonance imaging.

c LCS: lung cancer screening.

d CT: computed tomography.

e CTC: computed tomography colonography.

f *ICD*: *International Classification of Diseases*.

g CPT: current procedural terminology.

**Table 2.** The table shows examples of the 5 most common inquiries for each imaging-based cancer screening examination from May 1, 2022, to April 30, 2023. All data were obtained from Keyword tool (keywordtool.io) for worldwide searches performed in English.

	Example #1	Example #2	Example #3
Mammography			
Nearest screening locations	“mammography near me”	“mammogram without referral near me”	“mammogram near me now”
General inquires	“what is the mammography”	“why mammography is important”	“mammography versus mammogram”
Pain associated with screening	“is mammography painful”	“do mammograms hurt”	“what does a mammogram feel like”
Cancer imaging characteristics	“mammogramof breast cancer”	“mammography of breast cancer images”	“mammography of fibroadenoma”
Screening eligibility criteria	“mammography at what age”	“when should mammograms be done”	“mammography before 40”
Lung cancer screening			
CT^a^-specific inquiries	“lung cancer screening with low dose ct”	“low dose lung cancer screening”	“lung cancer detection using ct scan images”
General inquires	“lung cancer screening tests”	“what is a lung cancer screening”	“lung cancer screening for smokers”
Artificial intelligence and deep learning in LCS^b^	“lung cancer detection using image processing”	“lung cancer detection using deep learning”	“lung cancer detection using machine learning”
Screening eligibility criteria	“lung cancer screening ages”	“criteria for lung cancer screening”	“who should be screened for lung cancer”
Nearest screening locations	“lung cancer screening near me”	“mobile lung cancer screening near me”	“private lung cancer screening near me”
CT colonography			
CT-specific inquiries	“what is a ct colonography”	“ct colonography with contrast”	“ct colonography without contrast”
Screening procedural details	“ct colonography procedure”	“does ct colonography use contrast”	“how is ct colonography performed”
Imaging modality comparison (colonoscopy versus CTC^c^)	“ct colonography versus colonoscopy cost”	“ct colonography versus colonoscopy sensitivity”	“is ct colonography as good as colonoscopy”
Prescreening procedural preparation	“what is the prep for a ct colonography”	“what is the prep for a ct colonography nhs”	—^d^
Coding (*ICD*^e^/CPT^f^)	“ct colonography cpt code”	“ct colonography cpt”	—

a CT: computed tomography.

b LCS: lung cancer screening.

c CTC: computed tomography colonography.

d Not available.

e *ICD*: *International Classification of Diseases*.

f CPT: current procedural terminology.

## Discussion

### Principal Findings

#### Overview

Our study revealed much higher Google search volumes for MG topics over the past 2 decades than for LCS and CTC. The average search frequency for LCS was only approximately one-tenth of that for MG, and CTC search volume was only one–twenty-fifth of MG search volume. LCS average search volumes increased from 2011 to 2023, while searches for MG and CTC plateaued. MG-related topics had the highest total monthly search volume, followed by those related to LCS and CTC. Frequently searched topics varied across modalities and included nearby screening locations, procedural details or associated pain, and eligibility criteria.

MG consistently exhibited higher levels of search interest compared with LCS or CTC, possibly reflecting the longer history of MG and various longstanding initiatives focused on breast screening and women’s health [25,26]. Despite the higher absolute mortality for lung cancer than for breast cancer globally, search volumes for topics related to LCS have remained much lower than those for MG, although LCS search volumes have experienced an uptrend since 2011, when the National Lung Screening Trial was published [3]. MG was first established in 1913 and fully adopted in the 1970s [25], in contrast to low-dose CT for LCS, which gained widespread recognition in 2011. The early peak of interest in LCS in the 2004‐2006 period coincides with attention generated by early CT LCS programs, followed by a decline in interest until 2011 [27,28]. The relative search volumes for CTC have been persistently low over the last 2 decades, especially compared with MG (2%). While CTC is recognized by the national medical societies and formal guidelines of several countries as a viable screening option [29-31], other organizations have raised concerns regarding the strength of evidence supporting CTC or cost-effectiveness compared with colonoscopy [32,33]. The low search volumes might reflect a lack of awareness or desire for this screening option among the public.

Our topic analysis of recent volumes for queries related to image-based screening techniques showed both similarities and differences across imaging modalities. For example, in the case of MG, a substantial portion of searches (60,850/253,810, 24%) involved the nearest screening center, a topic with substantial although lower percentages of search volumes for LCS (750/11,150, 7%) and CTC (230/5590, 4%), highlighting a possible target of increased or more effective publicity. A relatively high volume of searches related to procedural aspects of CTC (1380/5590, 25%) may suggest a relative unfamiliarity with details of this specific imaging modality. A common topic of searches was potential procedural discomfort or pain in MG (28,970/253,810, 11%), with a lower percentage for CTC (220/5590, 1%), suggesting an opportunity for providers to better address procedural comfort that might otherwise decrease screening uptake. Cost or insurance coverage for screening was in the top 10 most-searched topics for all 3 screening modalities but comprised a minority of searches in terms of percentage; although cost and coverage pose concerns for some individuals, it is notable that several other topics, such as the nearest screening locations, procedural pain, and eligibility criteria, had on average 2- to 3-fold higher search volumes. AI and deep learning were common search topics for LCS (1210/11,150, 11%) but were not common queries for MG or CTC; this is somewhat surprising, as AI in the form of computer-aided detection is commonly used in MG.

#### Comparison to Prior Work

Previous research has examined internet search trends for cancer screening examinations. Snyder et al [20] examined Google search volume trends for cancer screening terms during the first stages of the COVID-19 pandemic, finding a temporary decline in searches for terms related to MG, LCS, colonoscopy, and pap smear. Rosenkrantz and Prabhu [22] performed a Google Trends analysis of the relative frequency of Google searches for MG, LCS, CTC, and prostate magnetic resonance imaging from 2004 to 2014, finding a slight progressive decline over the decade in searches for MG, a decrease from 2004 to 2010 in searches for LCS, followed by a persistent increase beginning in 2011, and an overall decade-long decline in searches for CTC; these findings are consistent with our analysis, although we show that searches for LCS continued to rise from 2014 to 2023 and that searches for MG and CTC have continued to plateau. Our analysis also has the advantage of comparing both the relative and absolute volumes of searches across modalities (MG, LCS, and CTC) instead of relying solely on relative search volumes, showing that searches related to MG greatly exceeded those related to LCS and CTC.

A variety of other methods have been used to assess public interest in and knowledge of cancer screening, including interviews, focus group discussions, questionnaires, and news coverage analysis [10,34,35]. Raju et al’s [34] survey of LCS-eligible individuals who chose not to participate in screening found that 19% of individuals had concerns about the distance to the screening site, and 14% of individuals had concerns about insurance coverage, recalling some of the frequent search topics for LCS in our study. In a Google News analysis of news coverage of MG from 2006 to 2015 [21], the most frequently covered topics included screening MG controversies (29%), new breast imaging technology (23%), imaging of dense breasts (11%), and public screening initiatives (11%), topics that were not among the most common MG searches in our study.

#### Strengths and Limitations

To our knowledge, our study is the first to use internet search engine data to gauge both general and topic-level public interest across imaging-based screening modalities by providing both relative search frequencies and estimates of absolute search volumes. We examined 2 decades of Google search volumes across 3 key imaging-based cancer screening examinations and documented the most common themes of recent related search queries.

Our study had several limitations. First, there are limitations inherent in any method of search volume estimation. Google Trends reports search data in relative terms and may report a value of 0 when search frequencies are low. For topic analysis, we captured data representing a snapshot of queries over a year, while queries may change over time. Search volumes were also estimated through a proprietary tool that uses Google autocomplete as a proxy for search volumes, as actual absolute search volumes are not available from Google Trends. However, our goal was not to measure exact search volumes but rather to analyze and compare trends across modalities and to discern the topics of the most frequent searches. Second, the analysis of thematic categories is intrinsically subjective; we attempted to mitigate this by using 2 observers, with final decisions rendered by consensus. Third, we analyzed worldwide Google searches in English and did not perform a country or region-specific analysis; while such comparative analyses are potentially of interest because of differences in screening guidelines and behavior across countries, our main objectives were to provide a general and comprehensive analysis of search interest across modalities and to determine the main topics of search queries. Future studies may incorporate comparisons of search queries across countries. Finally, internet search volumes may not directly translate to individual concerns, real-world behaviors, or screening uptake; however, individuals frequently turn to the internet as a source of medical information [16], and internet searches have often been used as a proxy of public interest in a wide variety of health-related topics [17-20,22].

### Conclusions

In conclusion, MG has generated consistently higher Google search volumes over the past 2 decades than LCS and CTC, but search interest in LCS has been on an upward trend since 2011. Frequently searched topics varied across modalities and included nearby screening locations, procedural details or associated pain, and eligibility criteria. These search trends might inform the development of communication strategies related to screening and aid in addressing frequently asked questions from the public.

## Abbreviations:

AI: artificial intelligence
CT: computed tomography
CTC: computed tomography colonography
LCS: lung cancer screening
MG: mammography
